# A Comparative Study on Three Analytical Methods for the Determination of the Neurotoxin BMAA in Cyanobacteria

**DOI:** 10.1371/journal.pone.0036667

**Published:** 2012-05-03

**Authors:** Elisabeth J. Faassen, Frits Gillissen, Miquel Lürling

**Affiliations:** Department of Environmental Sciences, Wageningen University, Wageningen, The Netherlands; Royal Netherlands Institute of Sea Research (NIOZ), The Netherlands

## Abstract

The cyanobacterial neurotoxin β-*N*-methylamino-L-alanine (BMAA) has been considered a serious health threat because of its putative role in multiple neurodegenerative diseases. First reports on BMAA concentrations in cyanobacteria were alarming: nearly all cyanobacteria were assumed to contain high BMAA concentrations, implying ubiquitous exposure. Recent studies however question this presence of high BMAA concentrations in cyanobacteria. To assess the real risk of BMAA to human health, this discrepancy must be resolved. We therefore tested whether the differences found could be caused by the analytical methods used in different studies. Eight cyanobacterial samples and two control samples were analyzed by three commonly used methods: HPLC-FLD analysis and LC-MS/MS analysis of both derivatized and underivatized samples. In line with published results, HPLC-FLD detected relatively high BMAA concentrations in some cyanobacterial samples, while both LC-MS/MS methods only detected BMAA in the positive control (cycad seed sarcotesta). Because we could eliminate the use of different samples and treatments as causal factors, we demonstrate that the observed differences were caused by the analytical methods. We conclude that HPLC-FLD overestimated BMAA concentrations in some cyanobacterial samples due to its low selectivity and propose that BMAA might be present in (some) cyanobacteria, but in the low µg/g or ng/g range instead of the high µg/g range as sometimes reported before. We therefore recommend to use only selective and sensitive analytical methods like LC-MS/MS for BMAA analysis. Although possibly present in low concentrations in cyanobacteria, BMAA can still form a health risk. Recent evidence on BMAA accumulation in aquatic food chains suggests human exposure through consumption of fish and shellfish which expectedly exceeds exposure through cyanobacteria.

## Introduction

The neurotoxic amino acid β-*N*-methylamino-L-alanine (BMAA) has been linked to neurodegenerative diseases as Alzheimer's disease (AD), Parkinson's disease (PD) and amyotrophic lateral sclerosis (ALS) [1]. BMAA was first identified in 1967 in the seeds of the cycad *Cycas micronesica* [2] in a survey on the cause of the high incidence of ALS, PD and dementia on Guam [3]. The possible etiological role of BMAA in this neurodegenerative disease was at first disputed [4], but was recently resurrected by the discovery of high concentrations of BMAA in the protein associated fraction of the cycad seeds and its biomagnification in the Guamanian food chain [5]–[8]. Furthermore, the finding of BMAA in the brains of people who had died with AD, ALS or PD outside Guam pointed towards a wider occurrence of BMAA [9], [10]. The detection of BMAA in the cyanobacterium *Nostoc sp.* that lives in symbiosis with the cycads [6] prompted the screening of cyanobacteria from all over the world for BMAA [11]–[23].

First reports on BMAA concentrations in cyanobacteria were alarming: high concentrations of BMAA were detected in nearly all tested free living laboratory strains [11], field isolates [16], field samples [13] and symbiotic species [11] (Table 1). In contrast, all but one [21] later studies could not reproduce these first results; BMAA was either not detected in cyanobacteria (e.g. [17], [18]), detected in some, yet not all, samples [23] or detected in all samples, but at very low concentrations [14]. The suggestion that BMAA might have been confused with its structural isomer α,γ-diaminobutyric acid (DAB) in the early studies [17] could be refuted [24].

**Table 1:** Overview of studies that analyzed more than eight samples of free living cyanobacteria for BMAA.

Tested samples	Fraction samples positive for BMAA	BMAA concentration in positive samples^a^	Analytical quantification method	Derivatization method	Reference
(n)	(-)	(µg/g DW)			
8	1.00	402 (190–1110)	CE	None	[21]
30	0.97	968 (10–6721)	HPLC-FLD	AccQ®-Tag	[11]
12	1.00	103 (8–287)	HPLC-FLD	AccQ®-Tag	[13]
27	0.96	129 (0.1–2757)	GC-MS	EZ:faast™	[16]
21	1.00	6.6*10^−3^ (1*10^−3^–15*10^−3^)	LC-MS/MS	AccQ®-Tag	[14]
20	0.95	1.35 (0.05–10.7)	LC-MS	EZ:faast™	[22]
21	0.42	13 (4–42)	LC-MS/MS	None	[23]
36	0.00	-	LC-MS/MS	None	[18]
30	0.00	-	LC-MS/MS	None	[17]

a BMAA concentration is the sum of the free and protein associated concentrations. Values are averages, followed by minimum and maximum concentrations between brackets.

Nonetheless there is still little consensus on BMAA concentrations in cyanobacteria. Cyanobacteria are ubiquitous and multiple routes of human exposure to cyanobacteria and their toxins exist [25]. It is therefore very important for human risk assessment to find the cause of the discrepancy in published results on BMAA concentrations in cyanobacteria.

Several factors underlie the different studies. Researchers have used different samples, different sample treatments and different analytical methods [24]. However, the differences in results seem to be related to the analytical method used. High BMAA concentrations and high percentages of positives samples were found only in those studies that had used high performance liquid chromatography with fluorescence detection (HPLC-FLD), gas chromatography with mass spectrometry detection (GC-MS) or capillary electrophoresis (CE) for quantification (Table 1). On the other hand, studies that had used high performance liquid chromatography with tandem mass spectrometry detection (LC-MS/MS) for quantification either did not detect BMAA, or reported lower BMAA concentrations (Table 1). Therefore, we hypothesized that different analytical methods for the determination of cyanobacterial BMAA deviate in their results. To test the hypothesis, we analyzed a set of cyanobacterial and control samples with three analytical methods: HPLC-FLD, LC-MS/MS of derivatized samples and LC-MS/MS of underivatized samples. The observed differences in our study were comparable to the observed differences in literature and were caused by overestimation of BMAA concentrations by HPLC-FLD.

## Results

### Method validation

Before sample analysis, all three methods were validated. Results of method validation are shown in Table 2. LC-MS/MS analysis of samples was performed with deuterium labeled BMAA (D_3_BMAA) as an internal standard. However, the validation of both LC-MS/MS methods was performed without correction for the internal standard to make comparison with the HPLC-FLD method possible. HPLC-FLD response was linear up to a concentration of 1000 µg/l, while both LC-MS/MS responses were linear up to 500 µg/l (Table 2). When corrected for the response of D_3_BMAA however, the LC-MS/MS methods showed a broader range of linearity (Figure S1). For all three methods, the fit of the regression line was good (r^2^>0.999).

**Table 2:** Validation results of the three used methods, both LC-MS/MS methods are validated without correction for the internal standard D_3_BMAA.

		HPLC-FLD	LC-MS/MS derivatized	LC-MS/MS underivatized
**Linearity** a				
Lowest concentration	µg/l	15	5	7.5
Highest concentration	µg/l	1000	500	500
Number of concentrations in tested range	-	6	8	9
r^2^	-	0.999	0.999	0.999
**Detection and quantification limits**				
LOD calibration standard	fmole/injection	68	85	106
LOQ calibration standard	fmole/injection	102	85	317
LOD sample extract	µg/g	*	1.0	0.4
LOD sample hydrolyzed	µg/g	40	10.0	1.6
LOQ sample extract	µg/g	*	1.0	0.4
LOQ sample hydrolyzed	µg/g	120	10.0	1.6
**Precision**				
Intraday precision (n = 6), response	Relative SD (%)	2.8	3.0	0.7
Intraday precision (n = 6), RT	Relative SD (%)	0.0	0.1	0.1
Interday precision (n = 12), response	Relative SD (%)	4.6	5.0	1.9
Interday precision (n = 12), RT	Relative SD (%)	0.1	0.0	0.2
Inter workup (n = 12) extract, response	Relative SD (%)	8.6	6.7	7.1
Inter workup (n = 12) extract, RT	Relative SD (%)	0.1	0.0	0.4
Inter workup (n = 12) hydrolyzed, response	Relative SD (%)	17.0	10.6^b^	6.1
Inter workup (n = 12) hydrolyzed, RT	Relative SD (%)	0.1	0.1^b^	0.1

LOD: limit of detection, LOQ: limit of quantification, RT: retention time,

a each concentration is injected in triplicate,

b n = 11,

* not determined (see text).

Detection and quantification limits of calibration standards were within the same range for all three methods. For the LC-MS/MS methods, the limit of detection (LOD) often equalled the limit of quantification (LOQ). This is possible because for these methods, LOD is defined as the lowest concentration where the signal-to-noise (S/N) ratio of all product ions is at least 3∶1 and the ratio of the qualifier ion(s) to the quantifier ion is within a 20% relative range. The conditions for the ratio of the qualifier ions to the quantifier ion or for the S/N of the qualifier ions are often only met at a S/N ratio of the quantifier ion of 10∶1, in which case the criteria for LOQ are also met. Chromatograms of LODs in samples are shown in Figure S2. Detection limits in samples are higher for both derivatized methods than for underivatized LC-MS/MS analysis. This is due to the dilution during derivatization (both extracts and hydrolyzed samples) and the extra dilution that is needed in the hydrolyzed samples to ensure effective derivatization [26], [27]. A single LOD or LOQ of free BMAA in samples could not be determined for the HPLC-FLD method. The extracts of cyanobacterial samples showed many low peaks around the retention time of BMAA, which made a good estimation of the position of the baseline difficult. For each sample, the pattern of these peaks was different, so no universal LOD or LOQ could be derived. This problem did not occur in the hydrolyzed samples, these chromatograms all showed fewer but higher peaks, with a better definable baseline for BMAA. Because baseline variation was higher for HPLC-FLD analysis than for LC-MS/MS analysis of derivatized samples, detection and quantification limits of the latter method in samples were lower. Underivatized LC-MS/MS was the most sensitive method for analysis of both extracted and hydrolyzed samples.

Interday and intraday precision was good for all three methods, underivatized LC-MS/MS analysis was most precise. Inter workup of hydrolyzed samples analyzed by HPLC-FLD showed an unexplainable high variation. Retention times in HPLC-FLD analysis were sensitive to variations in the buffer solution. This resulted in retention time differences between runs of maximum 0.3 min. Within runs, retention times were stable (Table 2).

Of the samples that were spiked before extraction, between 83.6 and 86.8% of the expected signal was recovered. Samples that were spiked before hydrolysis showed a lower recovery: between 46.7 (HPLC-FLD) and 69.3% (underivatized LC-MS/MS, Table 3).

**Figure 1:**
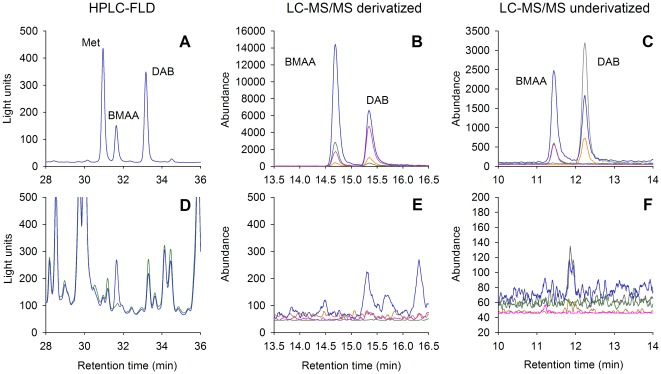
Chromatograms of the three analytical methods showing calibration standards and an extracted cyanobacterial sample. Panels A–C show calibration standards, panels D–F show the extracted *Anabaena* field scum. The green line in panel D represents the unspiked cyanobacterial sample, the blue line indicates the same sample, but spiked with BMAA before extraction. Colored lines in panels B and E represent the transitions of ions with a mass-to-charge ratio (*m/z*) of 459 to *m/z* 171 (blue), 119 (green), 145 (pink) and 315 (orange). Colored lines in panels C and F represent the transitions of *m/z* 119.1 to *m/z* 102.1 (blue), 88 (pink), 76 (green), 101 (gray) and 74 (orange). Transitions for D_3_BMAA are not shown.

All three methods separated BMAA from its isomer DAB (Figure 1).

### BMAA in samples

Analysis of the same samples by the three methods yielded different results (Table 4). Both LC-MS/MS methods only indicated BMAA in the positive control, the sarcotesta of the cycad seed. HPLC-FLD however indicated BMAA not only in the cycad seed, but also in three of the eight cyanobacterial samples.

Of the three methods used, HPLC-FLD has the highest detection limit for BMAA in samples. All BMAA concentrations as determined by HPLC-FLD were therefore far above the detection limit of both LC-MS/MS methods (Tables 2 and 4). If samples indeed contained BMAA concentrations as high as indicated by HPLC-FLD, both LC-MS/MS methods should have detected BMAA as well.

Free BMAA concentrations in the cycad seed sarcotesta were in the same range for all three methods. Total BMAA was also detected in the cycad seed by all three methods, but was below the LOQ for HPLC-FLD (Table 4).

## Discussion

We clearly showed that BMAA concentrations in some cyanobacterial samples varied depending on the analytical method used. The three methods only indicated similar BMAA concentrations in the positive control, the sarcotesta of a cycad seed. Since we have used the same samples and employed identical sample treatments, sample origin and treatment could be eliminated as possible causal factors [24] of the observed differences. The differences in BMAA concentrations can therefore only be attributed to the analytical methods, and are roughly in line with the observed discrepancy in published results (Table 1).

HPLC-FLD identified BMAA in three out of eight cyanobacterial samples, while both LC-MS/MS methods did not detect any BMAA in cyanobacteria. These differences are most likely due to the low selectivity of the HPLC-FLD. HPLC-FLD is less selective than both LC-MS/MS methods, it has only two selection criteria: retention time and fluorescence signal. Because BMAA does not have fluorescent properties, derivatization with the fluorescent AccQ®-Tag was necessary for detection by HPLC-FLD. Both retention time and fluorescence signal are properties of the derivative of an analyte, instead of the analyte itself and any compound that reacts with the AccQ®-Tag gives the same fluorescence signal after derivatization. The AccQ®-Tag reacts with primary and secondary amino groups [28], [29], which means that it reacts with all amino acids and other amino group containing compounds. There are hundreds of naturally occurring amino acids [30], so there is always a chance that a derivatized compound other than BMAA has the same or similar retention time as the BMAA derivative. If such a compound is present in a sample, it leads to misidentification and subsequent overestimation of BMAA concentrations in that sample. HPLC-FLD is therefore an uncertain method for amino acid analysis in complex biological matrices that contain non-protein amino acids or other compounds with an amino group [29], especially when the analyte is present in low concentrations.

LC-MS/MS is a more selective method than HPLC-FLD because it has four selection criteria: retention time, mass-to-charge ratio (*m/z*) of the precursor ion (the charged ‘original’ molecule), *m/z* of the product ions after collision induced dissociation and the ratio between the abundance of the product ions. The chance of compound misidentification by LC-MS/MS is therefore much smaller than by HPLC-FLD. In our study, LC-MS/MS peaks were only identified as BMAA when all four criteria were met.

**Table 3:** Recovery (%) of extraction and hydrolysis, analyzed by the three different methods.

	Extraction			Hydrolysis		
	average	SD	n	average	SD	n
HPLC-FLD	86.8	10.1	12	46.7	8.5	12
LC-MS/MS derivatized^a^	83.6	5.5	12	68.6	6.8	11
LC-MS/MS underivatized^a^	85.5	5.9	12	69.3	4.2	12

a recovery is calculated for D_3_BMAA.

The discrepancy in the results could not be caused by other factors like method sensitivity or difference in sample treatment (derivatization versus underivatized analysis). In our study, the least sensitive method (HPLC-FLD) gave more positive results than the more sensitive LC-MS/MS methods. Quantification by LC-MS/MS was reliable because we used D_3_BMAA as an internal standard in all samples [18], resulting in unbiased estimates of BMAA concentrations in samples, also in low concentrations (Figure S3). The observed differences in results can therefore not be explained by differences in method sensitivity. Also derivatization cannot explain the differences in results, because both derivatized methods (HPLC-FLD and LC-MS/MS analysis of derivatized samples) varied in their outcome. It has been suggested that (underivatized) HILIC LC-MS/MS analysis is less suitable for BMAA detection than LC-MS/MS analysis after derivatization [24], but our study does not support this hypothesis as underivatized LC-MS/MS analysis was in our case more precise and more sensitive for samples than derivatized LC-MS/MS analysis (Table 2). Furthermore, the warning that the signal of methionine methylsulphonium might interfere with that of BMAA in underivatized LC-MS/MS analysis [31] is unnecessary, because this compound has a different molecular weight than BMAA and its signal will therefore not be picked up in MRM analysis. We conclude that the most likely cause of the differences in our experiment is that HPLC-FLD has misidentified another amino containing compound as BMAA in some samples and consequently has overestimated BMAA concentrations in these samples.

From our study, it cannot be determined which compound has mistakenly been identified as BMAA by HPLC-FLD analysis. While attempts have been made to exclude compounds from being possibly interfering in various methods of BMAA analysis [24], [31], these studies only focus on a few compounds, mostly diamino acids. The possible similarity of the fragmentation pattern of diamino acids with that of BMAA makes these compounds likely candidates for interference with mass spectrometry analyses. The compounds tested in these two studies did not interfere with the BMAA signal in most tested methods, only one compound co-eluted in an UHPLC-UV/MS method [31]. However, the list of possible interfering compounds in HPLC-FLD analysis is much larger and also includes compounds with only one amino group. These two studies can therefore not be used to identify possibly interfering compounds in previously performed HPLC-FLD analyses. Furthermore, different chromatographic conditions can result in different interfering compounds, which means that the compound that has mistakenly been identified in our study, can be another compound than the one that has interfered in other studies.

The average BMAA concentrations found by HPLC-FLD in this study are lower than concentrations found in free living cyanobacteria by previous studies that used HPLC-FLD for quantification [11], [13] (Table 1). Furthermore, in our study BMAA was identified in only three of the eight tested cyanobacterial samples, while the other HPLC-FLD studies report presence of BMAA in nearly all tested cyanobacteria. Again, this is most likely due to the differences in chromatographic conditions between the studies. Although presence of BMAA has been confirmed by LC-MS/MS in the early HPLC-FLD based studies, BMAA concentrations determined by these LC-MS/MS analyses have not been reported [11], [13]. It is therefore unknown whether the concentrations found by HPLC-FLD matched the concentrations found by LC-MS/MS in these studies. Also in the only other study, so far, that compared different analytical methods, BMAA concentrations based on LC-MS(/MS) analyses were not reported [32].

Certainly, variations in BMAA concentrations found in different studies may not only be caused by the use of different analytical methods. BMAA concentrations may also differ as a result of the origin or growth conditions of the cyanobacteria, which is the case for most other cyanobacterial toxins [33]. An attempt has been made to determine conditions under which cyanobacteria produce BMAA [34], but much work is still needed to understand BMAA production. Origin and growth conditions cannot explain the incongruity observed in our study because we analyzed the same material with different analytical methods. So, although the high BMAA concentrations measured in the early studies may indeed have resulted from samples that contained high amounts of BMAA, it is more plausible that they are an artifact of the HPLC-FLD method. Our results suggest that BMAA is not present in high concentrations in cyanobacteria. Presence of lower concentrations of BMAA (low µg/g DW or ng/g DW) in (some) cyanobacteria is more likely and could also explain why BMAA is detected by some selective methods with high sensitivity [14], [15] and is not detected [17]–[19] or detected only in a number of samples [23] by studies that have a lower sensitivity.

Our study only focused on three analytical methods and can therefore not explain other discrepancies in published BMAA concentrations than those between HPLC-FLD and (derivatized or underivatized) LC-MS/MS analysis. More work is for instance needed to explain the differences in concentrations found in cyanobacterial field isolates from similar regions and grown under similar condition that were analyzed by the same group by GC-MS and LC-MS [16], [22]. Also the high concentrations found by CE analysis [21] are interesting, even though the authors of this last manuscript acknowledge that the selectivity of their method is low. In general, comparison of the quantitative results of different studies is hampered by the absence of recovery and validation data in many publications.

Even if BMAA concentrations in cyanobacteria are low, BMAA can still pose a threat to human health. First, BMAA has the ability to accumulate in food chains. In the Baltic sea, BMAA concentrations in zooplankton, shellfish and bottom-dwelling fish species are up to 200 fold higher than in the local cyanobacteria [14]. Moreover, laboratory studies have shown that the zooplankton species *Daphnia magna* is able to take up BMAA from its surrounding medium, thereby bioconcentrating BMAA up to 3800 times [35]. Presence of BMAA in the aquatic food chain means that people are not only exposed to BMAA by direct contact with cyanobacteria, but also through food. Exposure through food may extent over a larger area and a larger period of time than exposure through cyanobacteria. The dose of BMAA obtained through food might therefore exceed the dose obtained directly through cyanobacteria. Second, in addition to its own neurotoxicity, BMAA can also enhance the effect of other neurotoxins [36]. The additive effect of BMAA with other cyanobacterial neurotoxins has not been evaluated yet, but BMAA sometimes occurs simultaneously with the neurotoxins DAB [23], anatoxin-a and saxitoxin [13] and synergistic toxicity cannot on forehand be excluded.

We conclude that in our study HPLC-FLD overestimated BMAA concentrations in some cyanobacterial samples due to its low selectivity. Cyanobacterial BMAA concentrations seem to be overestimated in some previous studies as well and are more likely to be in the low µg/g DW or even in the ng/g DW range than in the high µg/g DW range as sometimes reported. We therefore recommend to only use selective and sensitive analytical methods like LC-MS/MS for BMAA analysis. Although possibly present in low concentrations in cyanobacteria, presence of BMAA in the aquatic food chain and possible synergistic effects with other cyanobacterial neurotoxins still urge for investigation on the risk of BMAA for human health.

## Materials and Methods

Eight cyanobacterial samples (four scum samples from the field and four laboratory strains), a negative control (a green alga) and a positive control (sarcotesta of a cycad seed) were prepared for analysis of free and total BMAA. The sample treatments were the ones that are most often applied: trichloroacetic acid extraction for analysis of free BMAA and acid hydrolysis for total BMAA. All sample treatments were performed in nine fold. Six replicates of each fraction were then derivatized using AccQ®-Tag and analyzed by HPLC-FLD (n = 3) or LC-MS/MS (n = 3). The other three replicates were analyzed by LC-MS/MS without derivatization. Prior to extraction or hydrolysis, deuterium labeled BMAA was added to the samples that were analyzed by LC-MS/MS as an internal standard. L-2-aminobutyric acid (AAbA) was added after extraction or hydrolysis to the samples that were analyzed by HPLC-FLD and was used as retention time reference. Validation of all methods was based on FDA guidelines [37], [38].

**Table 4:** Free and total BMAA concentrations (µg/g DW, average and SD, n = 3) in control and cyanobacterial samples as analyzed by three different methods.

	HPLC-FLD		LC-MS/MS derivatized		LC-MS/MS underivatized	
	Free	Total	Free	Total	Free	Total
**Controls**						
*S. obliquus* SAG 276/3a (neg)	n.d.	n.d.	n.d.	n.d.	n.d.	n.d.
Cycad seed sarcotesta (pos)	**18.2 (1.4)**	**d.**	**8.8 (3.0)**	**104.9 (4.5)**	**10.7 (2.9)**	**75.0 (10.8)**
**Field scums**						
*Anabaena*	**21.7 (3.1)**	n.d.	n.d.	n.d.	n.d.	n.d.
*P. rubescens*	**6.3 (0.8)**	n.d.	n.d.	n.d.	n.d.	n.d.
*Aphanizomenon*	n.d.	n.d.	n.d.	n.d.	n.d.	n.d.
*Microcystis*	n.d.	n.d.	n.d.	n.d.	n.d.	n.d.
**Lab strains**						
*C. raciborskii* CS-1	n.d.	n.d.	n.d.	n.d.	n.d.	n.d.
*Aph. flos-aquae* CCAP 1401/7	**56.2** a	**d.**	n.d.	n.d.	n.d.	n.d.
*An. flos-aquae* CCAP 1409/2A	n.d.	n.d.	n.d.	n.d.	n.d.	n.d.
*M. aeruginosa* NIVA CYA 228/1	n.d.	n.d.	n.d.	n.d.	n.d.	n.d.

n.d.: not detected, d.: detected but below limit of quantification,

a n = 2.

### Sample material

The control samples consisted of the green alga *Scenedesmus obliquus* SAG 276/3a (negative control) and the sarcotesta of a *Cycas micronesica* (Hill) seed (positive control). *S. obliquus* was cultured as in [35] and was harvested directly before sample preparation. The cycad seed was kindly provided by Chad Husby, Montgomery Botanical Centre, Miami, US and was stored at −20°C after picking. The cyanobacterial scum samples were collected in various lakes in The Netherlands in 2008 and 2009 and were dominated by either *Anabaena*, *Planktothrix rubescens*, *Aphanizomenon* or *Microcystis*. The cyanobacterial laboratory strains used were *Cylindrospermopsis raciborskii* CS-1, *Aphanizomenon flos-aquae* CCAP 1401/7, *Anabaena flos-aquae* CCAP 1409/2A and *Microcystis aeruginosa* NIVA CYA 228/1. The first three strains were grown in batch cultures on a modified WC medium [39] at room temperature at normal daylight and were harvested after a growth period of 20 to 25 days, while *Microcystis* was grown for 15 days at 20°C and in 45 µmol quanta m^−2^ s^−1^ light in a 16∶8 h light∶dark rhythm. All samples were lyophilized and stored at −20°C until preparation.

### Sample preparation

All lyophilized samples were homogenized and extracted or hydrolyzed. 5 mg of sample (0.5 mg for the cycad seed) was extracted for free BMAA at room temperature in the dark for two hours in 300 µl 0.1 N trichloroacetic acid (TCA). After the extraction, the sample was centrifuged and the supernatant was transferred. 300 µl 0.1 N TCA was then again added to the pellet and after vortexing and centrifugation the supernatant was pooled with the first supernatant and lyophilized. The dried supernatants were derivatized after dissolving them in 500 µl hot 20 mM HCl. Samples were derivatized by adding 60 µl buffer and 20 µl reagent (6-aminoquinolyl-N-hydroxysuccinimidyl carbamate, purchased as AccQ®-Tag, Waters) to 20 µl sample [26]. Dried supernatants for underivatized LC-MS/MS analysis were dissolved in 500 µl 65% acetonitrile, 35% Millipore water and 0.1% formic acid (v:v:v).

For total BMAA, 1 mg of lyophilized sample (0.5 mg for the cycad seed) was hydrolyzed in an hydrolysis/derivatization workstation (Eldex), using 6 N HCl liquid hydrolysis for 20 hours at 105°C in the absence of oxygen. After hydrolysis, samples for derivatized analysis were dissolved in 500 µl hot 20 mM HCl and subsequently diluted ten times in 20 mM HCl to obtain a protein concentration below 0.1 g/l ([26], [27], maximum protein content of cyanobacteria was estimated to be 50%). Derivatization procedure was the same as for the free fraction. Hydrolyzed samples for underivatized LC-MS/MS analysis were dissolved in 1 ml 65% acetonitrile, 35% Millipore water and 0.1% formic acid (v:v:v).

Deuterium labeled BMAA (D_3_BMAA, kindly provided by Johan Rosén, National Food Administration, Uppsala, Sweden and synthesized as in [18]) was added to the samples that were analyzed by LC-MS/MS prior to extraction or hydrolysis so the maximum concentration at the moment of analysis was 400 µg/l. L-2-aminobutyric acid (AAbA, Sigma-Aldrich) was added to the samples that were analyzed by HPLC-FLD after extraction or hydrolysis at a maximum concentration of 500 µg/l.

### Sample analysis

HPLC-FLD analysis was performed on an Agilent 1100 LC-FLD. Compounds were separated on a Nova-Pak C18 3.9×300 mm, 4 µm column (Waters). Eluent A consisted of 140 mM sodium acetate and 5.6 mM triethylamine in Millipore water, adjusted to pH 5.2 with phosphoric acid. Eluent B was acetonitrile and eluent C was Millipore water. The elution program was: 0 min 100% A; 7 min 90% A and 5.2% B; 10–20 min 84% A and 8.3% B; 23 min 75% A and 13% B; 38 min 65% A and 18.2% B; 40 min 40% A and 31.2% B; 42.5–52 min 52% B and 48% C; 55–65 min 100% A. Flow rate was 1 ml/min, injection volume 4 µl and column temperature 37°C. Excitation wavelength was 250 nm, emission wavelength was 395 nm.

LC-MS/MS analysis of the derivatized samples was performed on an Agilent 1200 LC and an Agilent 6401A QQQ. Compounds were separated on a Zorbax Eclipse AAA 4.6×75 mm, 3.5 µm column (Agilent) with mobile phases acetonitrile with 0.1% formic acid (v:v, eluent A) and Millipore water with 0.1% formic acid (v:v, eluent B). The following gradient was applied: 0 min 1% A; 4 min 2% A; 8 min 5% A; 18 min 10% A; 20–24 min 50% A; 24–38 min 0% A. Flow rate was 1 ml/min, injection volume 10 µl and column temperature 40°C. The LC-MS/MS was operated in positive mode with an ESI source, fragmentor voltage was 140 V. Nitrogen was used as the drying and collision gas. Quadrupole 1 was operated in unit mode and quadrupole 2 was operated in widest mode. BMAA was detected by the transitions mass-to-charge ratio (*m/z*) 459 to *m/z* 171 at 32 V collision energy, *m/z* 119 and *m/z* 145 (both 16 V). Ratio of the peak area of qualifier *m/z* 119 to the peak area of quantifier *m/z* 171 was 10%, ratio of the qualifier *m/z* 145 to *m/z* 171 was 14%. DAB was detected by the transitions *m/z* 459 to *m/z* 171 (28 V), *m/z* 145 and *m/z* 315 (both 12 V). Ratio of the qualifier *m/z* 145 to quantifier *m/z* 171 was 83% and ratio of the qualifier *m/z* 315 to *m/z* 171 was 8%. D_3_BMAA was detected by the transitions *m/z* 462 to *m/z* 171 (32 V), *m/z* 145 and *m/z* 122 (both 16 V). Ratio of the qualifier *m/z* 145 to quantifier *m/z* 171 was 13% and ratio of the qualifier *m/z* 122 to *m/z* 171 was 23%.

Underivatized samples were analyzed on the same LC-MS/MS equipment and with the same mobile phases as the derivatized samples. Compounds were separated on a 2.1×150 mm, 5 µm diameter ZIC®-HILIC column (Sequant) with a Direct-Connect™ Filter (Grace Alltech). Flow rate was 0.4 ml/min, injection volume 5 µl and column temperature 40°C. The following gradient was applied: 0–2 min 95% A; 4 min 65% A; 8–17 min 55% A; 17–23 min 95% A. Fragmentor voltage was 50 V and both quadrupoles were operated in unit mode. BMAA was detected by the transitions *m/z* 119.1 to *m/z* 102.1 (4 V), *m/z 88* and *m/z* 76 (both 8 V). Ratio of both qualifiers *m/z* 88 and *m/z* 76 to quantifiers *m/z* 102.1 was 21%. DAB was detected by the transitions *m/z* 119.1 to *m/z* 101 (4 V) and *m/z* 74 (8 V). Ratio of the qualifier *m/z* 76 to quantifier *m/z* 101 was 23%. D_3_BMAA was detected by the transitions *m/z* 122.1 to *m/z* 105.1 (4 V), *m/z 88* and *m/z* 76 (both 8 V). Ratio of qualifier *m/z* 88 to quantifier *m/z* 105.1 was 22%, ratio of *m/z* 76 to *m/z* 105.1 was 37%.

Calibration standards for the derivatized samples were prepared in 20 mM HCl and then derivatized, calibration standards for the underivatized samples were prepared in 65% acetonitrile, 35% Millipore water and 0.1% formic acid (v:v:v). Calibration standards for LC-MS/MS analysis contained BMAA, DAB (DAB Dihydrochloride, Sigma-Aldrich) and D_3_BMAA. Calibration standards for HPLC-FLD analysis contained BMAA, DAB, methionine (DL-Methionine, Fluka) and AAbA. BMAA concentrations in LC-MS/MS samples were determined by correcting the response of BMAA for the response of D_3_BMAA. BMAA concentrations analyzed by HPLC-FLD were calculated against the calibration curve and subsequently corrected for the recovery (see method validation).

### Method validation

To make comparison of the HPLC-FLD method with both LC-MS/MS methods possible, validation of both LC-MS/MS methods was performed without correction for the response of D_3_BMAA.

Linearity was determined by injecting a range of calibration standards in triplicate.

For both LC-MS/MS methods, limit of detection (LOD) in calibration standards was determined as the lowest injected concentration with a signal-to-noise (S/N) ratio of all product ions of at least 3∶1. Furthermore, the ratio of the qualifier ions to the quantifier ion should be within a 20% relative range of the expected value. Limit of quantification (LOQ) was defined as the lowest injected concentration with a S/N ratio of the quantifier ion of at least 10∶1. Furthermore, the ratio of the qualifier ions to the quantifier should again be within the accepted range, and the S/N ratio of the qualifier ions should at least be 3∶1. For HPLC-FLD analysis, LOD in calibration standards was defined as the lowest concentration of which the peak was clearly distinguishable from the background signal. LOQ was defined as the lowest concentration that was linear on the calibration curve. Detection and quantitation limits in samples were defined in the same way as for calibration standards. Limits in samples were determined by spiking an *Anabaena* scum sample with different BMAA concentrations prior to extraction or hydrolysis.

Intraday precision of response and retention time was determined by injecting the highest calibration standard (1000 µg/l) in six fold. For interday precision, calibration standards were injected again in six fold on a different day and the variation of all twelve injections was considered. Inter workup precision was determined by spiking an *Anabaena* scum sample in six fold before extraction or hydrolysis with either D_3_BMAA (both LC-MS/MS methods, 200 ng for free BMAA, 400 ng for underivatized total BMAA and 2000 ng for derivatized total BMAA) or BMAA (HPLC-FLD method, 750 ng for free BMAA and 9950 ng for total BMAA). The same sample treatment was repeated in six fold on another day and response was compared. The inter workup samples were also used for calculation of recovery, which is in this study defined as the percentage of the original signal that was recovered after sample preparation and analysis.

Since no reference material is available for BMAA, accuracy was not tested. Instead, a positive control sample (cycad seed sarcotesta) was included. The mass of cycad seed sarcotesta used for extraction and hydrolysis was lower than for the cyanobacterial samples, so the signal of the cycad seed would be close to the detection limits of the methods. Furthermore, recovery of all sample treatments was determined.

## Supporting Information

Figure S1 **LC-MS/MS BMAA calibration curves for derivatized analysis and underivatized analysis, corrected for D_3_BMAA.** Panel A shows the calibration curve for derivatized analysis, panel B for underivatized analysis. All concentrations are injected in triplicate, except 5 and 10 µg/l in panel A, these concentrations are injected once.
(TIF)

Figure S2 **Limits of detection (LODs) for BMAA in spiked** ***Anabaena*** **scum samples.** Panel A and B show HPLC-FLD signals, panel C and D show LC-MS/MS signals of derivatized samples and panel E and F show LC-MS/MS signals of underivatized samples. Panels C and E represent samples that are spiked with BMAA before extraction, panels B, D and F represent samples that are spiked before hydrolysis. No LOD could be defined for BMAA in extracted samples for HPLC-FLD analysis (see results in main text), panel A therefore shows an unspiked extracted field sample of *Planktothrix rubescens* with a low response at the retention time of BMAA (see also Table 4 in main text). Colored lines in panels C and D represent the transitions of ions with a mass-to-charge ratio (*m/z*) of 459 to *m/z* 171 (blue), 119 (green), 145 (pink) and 315 (orange). Colored lines in panels E and F represent the transitions of *m/z* 119.1 to *m/z* 102.1 (blue), 88 (pink), 76 (green), 101 (gray) and 74 (orange). Transitions for D_3_BMAA are not shown.
(TIF)

Figure S3 **Concentrations of BMAA in spiked** ***Anabaena*** **scum samples, analyzed by LC-MS/MS and corrected for D_3_BMAA.** Panel A shows extracted derivatized samples, panel B shows hydrolyzed derivatized samples, panel C shows extracted underivatized samples and hydrolyzed underivatized samples are shown in panel D. All samples are spiked before extraction or hydrolysis and are injected once.
(TIF)
