# Supplementary figures and images for: A Comparative Study on Three Analytical Methods for the Determination of the Neurotoxin BMAA in Cyanobacteria
Source: PLoS One. 2012 May 3;7(5):e36667. doi: 10.1371/journal.pone.0036667 (PMC3343013; doi:10.1371/journal.pone.0036667)

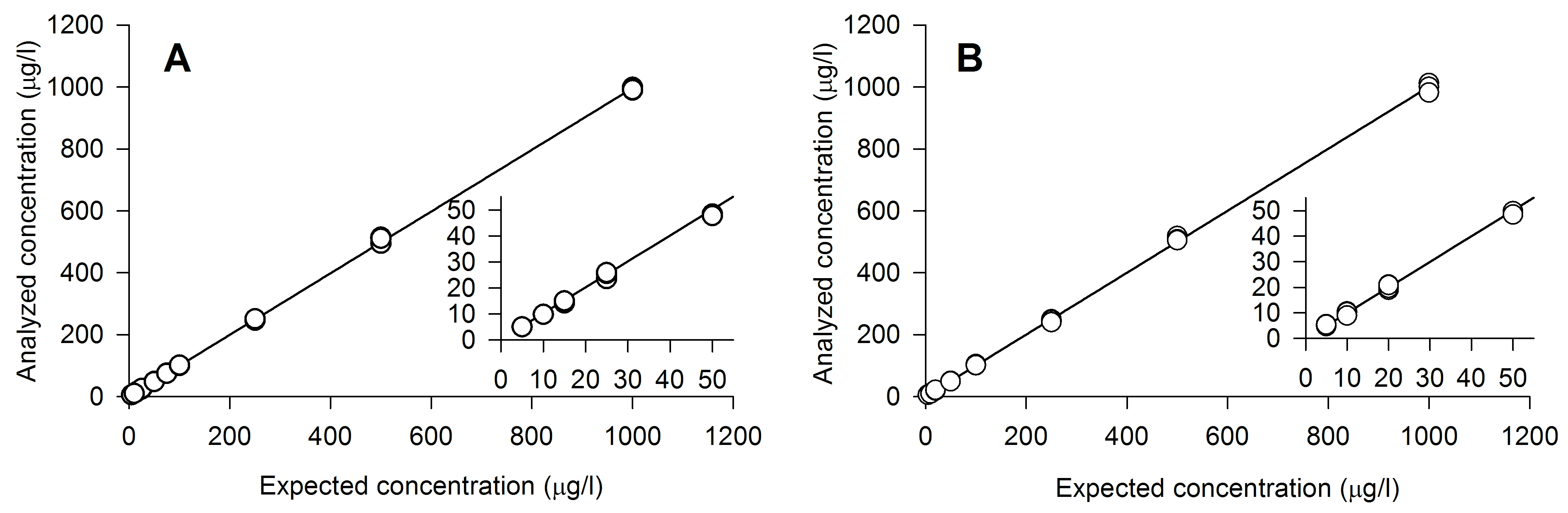

Supplement: Figure S1 — LC-MS/MS BMAA calibration curves for derivatized analysis and underivatized analysis, corrected for D3BMAA. Panel A shows the calibration curve for derivatized analysis, panel B for underivatized analysis. All concentrations are injected in triplicate, except 5 and 10 µg/l in panel A, these concentrations are injected once. (TIF) [file pone.0036667.s001.tif]

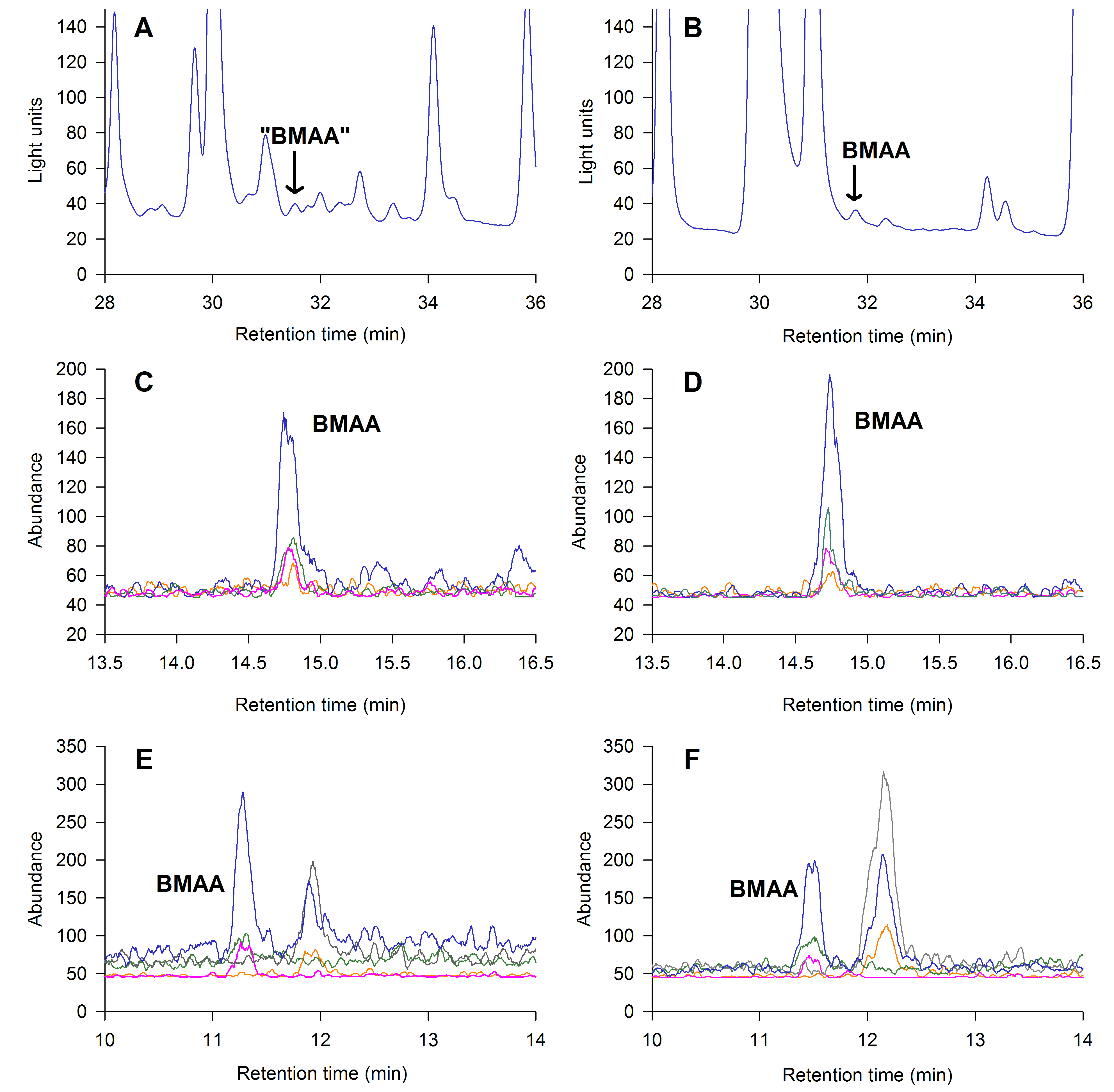

Supplement: Figure S2 — Limits of detection (LODs) for BMAA in spiked Anabaena scum samples. Panel A and B show HPLC-FLD signals, panel C and D show LC-MS/MS signals of derivatized samples and panel E and F show LC-MS/MS signals of underivatized samples. Panels C and E represent samples that are spiked with BMAA before extraction, panels B, D and F represent samples that are spiked before hydrolysis. No LOD could be defined for BMAA in extracted samples for HPLC-FLD analysis (see results in main text), panel A therefore shows an unspiked extracted field sample of Planktothrix rubescens with a low response at the retention time of BMAA (see also Table 4 in main text). Colored lines in panels C and D represent the transitions of ions with a mass-to-charge ratio (m/z) of 459 to m/z 171 (blue), 119 (green), 145 (pink) and 315 (orange). Colored lines in panels E and F represent the transitions of m/z 119.1 to m/z 102.1 (blue), 88 (pink), 76 (green), 101 (gray) and 74 (orange). Transitions for D3BMAA are not shown. (TIF) [file pone.0036667.s002.tif]

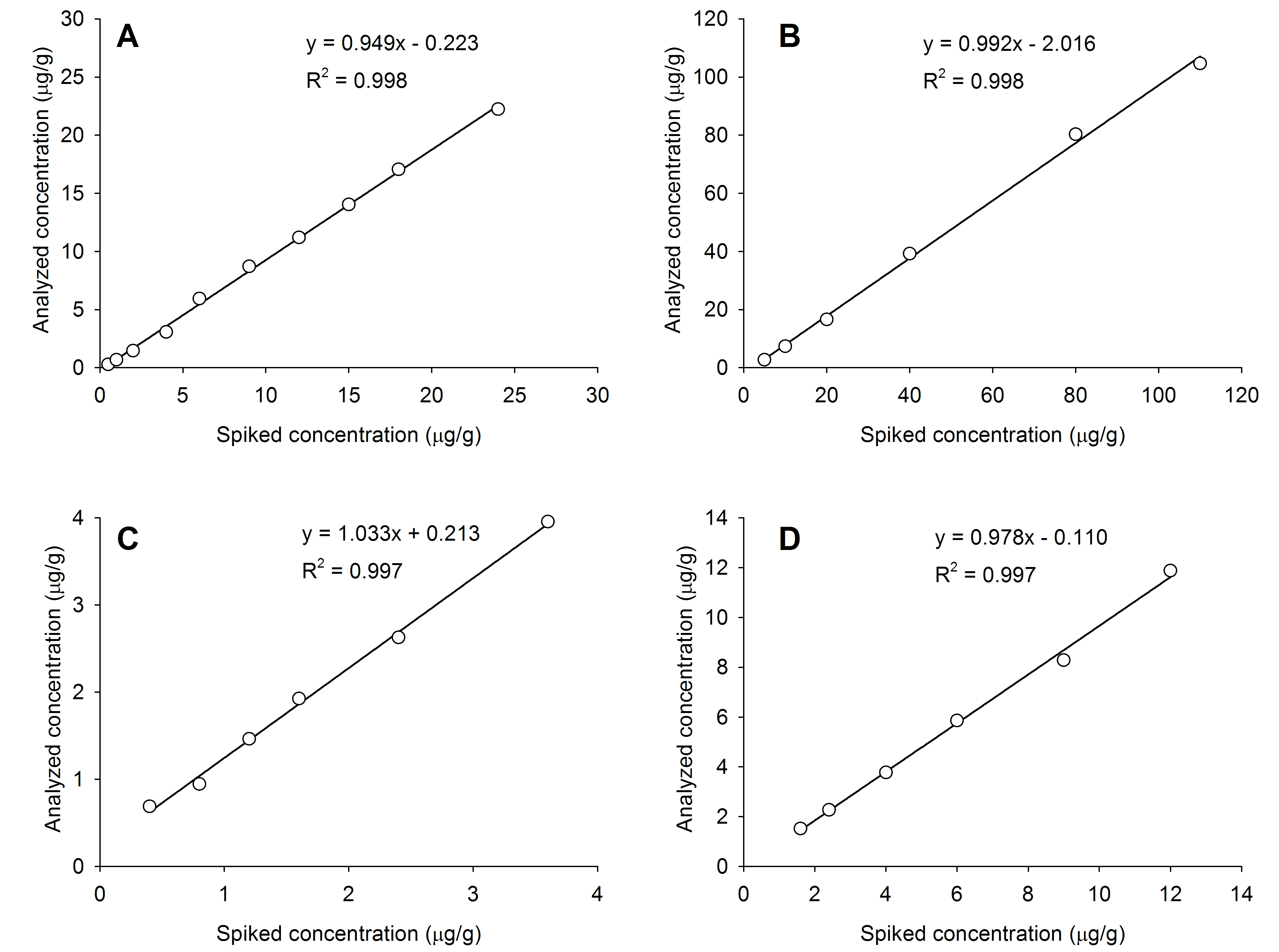

Supplement: Figure S3 — Concentrations of BMAA in spiked Anabaena scum samples, analyzed by LC-MS/MS and corrected for D3BMAA. Panel A shows extracted derivatized samples, panel B shows hydrolyzed derivatized samples, panel C shows extracted underivatized samples and hydrolyzed underivatized samples are shown in panel D. All samples are spiked before extraction or hydrolysis and are injected once. (TIF) [file pone.0036667.s003.tif]
